# NDRG4 sensitizes CRC cells to 5-FU by upregulating DDIT3 expression

**DOI:** 10.3892/ol.2022.13321

**Published:** 2022-05-10

**Authors:** Ruikai Li, Chenxiang He, Liangliang Shen, Shuai Wang, Yao Shen, Fan Feng, Jian Zhang, Jianyong Zheng

Oncol Lett 22: 782, 2021; DOI: doi.org/10.3892/ol.2021.13043

Following the publication of the above article, the authors have realized that the EdU images portrayed in Fig. 1D and the TUNEL images shown in Fig. 2C were derived from the incorrect folder during the process of compiling the figures.

The authors have consulted their original data, and have identified the correct data that should have been included in these figures. The corrected versions of Figs. 1 and 2, showing the correct data for the experiments shown in Fig. 1D and 2C, are shown on the next two pages. These errors did not have a significant impact on either the results or the conclusions reported in this paper. All the authors agree to the publication of this corrigendum, and are grateful to the Editor of *Oncology Letters* for allowing them the opportunity to publish this. Furthermore, they apologize to the readership for any inconvenience caused.

## Figures and Tables

**Figure 1.**
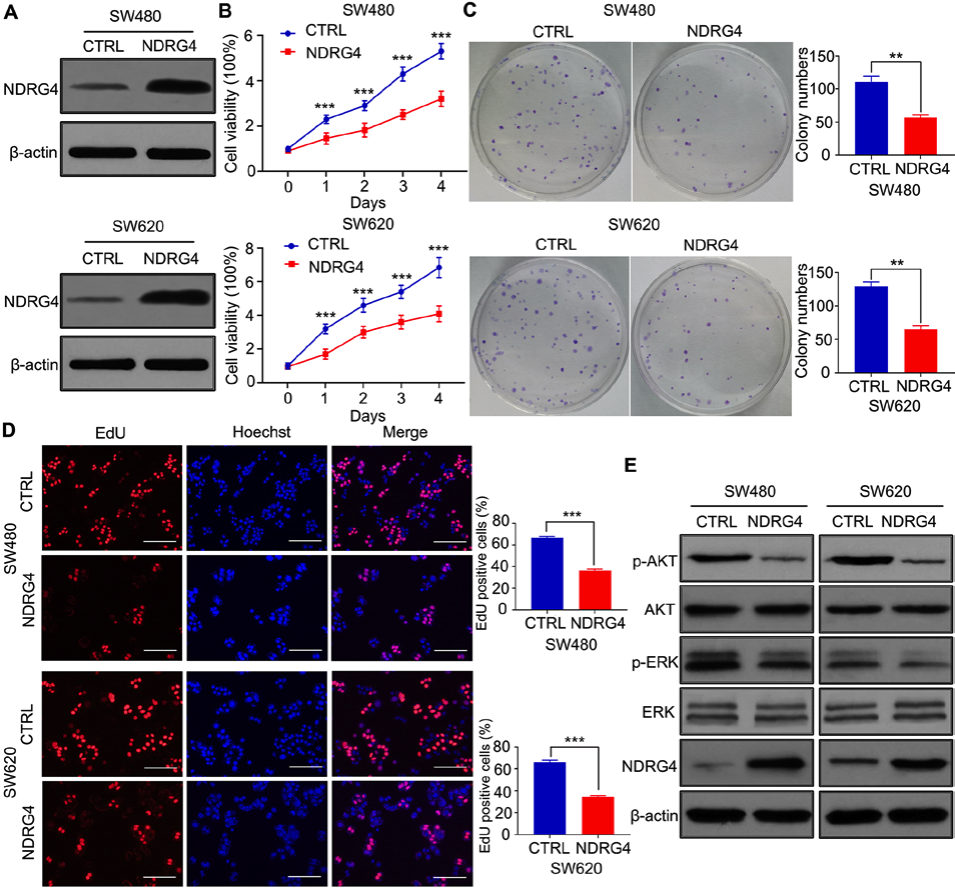
NDRG4 inhibits the proliferation of colorectal cancer cells. (A) Representative images of NDRG4 protein expression in SW480 and SW620 control and NDRG4-overexpressing cells detected by western blotting (n=3). (B) Viability of SW480 and SW620 control and NDRG4-overexpressing cells determined by MTT analysis. Statistical analysis by two-way ANOVA (n=5). (C) Representative images and statistical analyses of colony formation capacity of control and NDRG4-overexpressing SW480 and SW620 cells. Statistical analysis by Student's t-test (n=3). (D) Representative images and the statistical analyses of SW480 and SW620 control and NDRG4-overexpressing cell proliferation, determined by the EdU analysis. Scale bar, 150 μm. Statistical analysis by Student's t-test (n=3). (E) Representative images of p-AKT, AKT, p-ERK and ERK protein expression in SW480 and SW620 control and NDRG4-overexpressing cells detected by western blotting. Upper band of p-ERK represents phosphorylated ERK1, and the lower band represents phosphorylated ERK2. Upper band of ERK represents ERK1, and the lower band represents ERK2 (n=3). ***P<0.001 and **P<0.01 vs. CTRL. NDRG4, N-myc downstream-regulated gene 4; p-, phosphorylated; CTRL, control.

**Figure 2.**
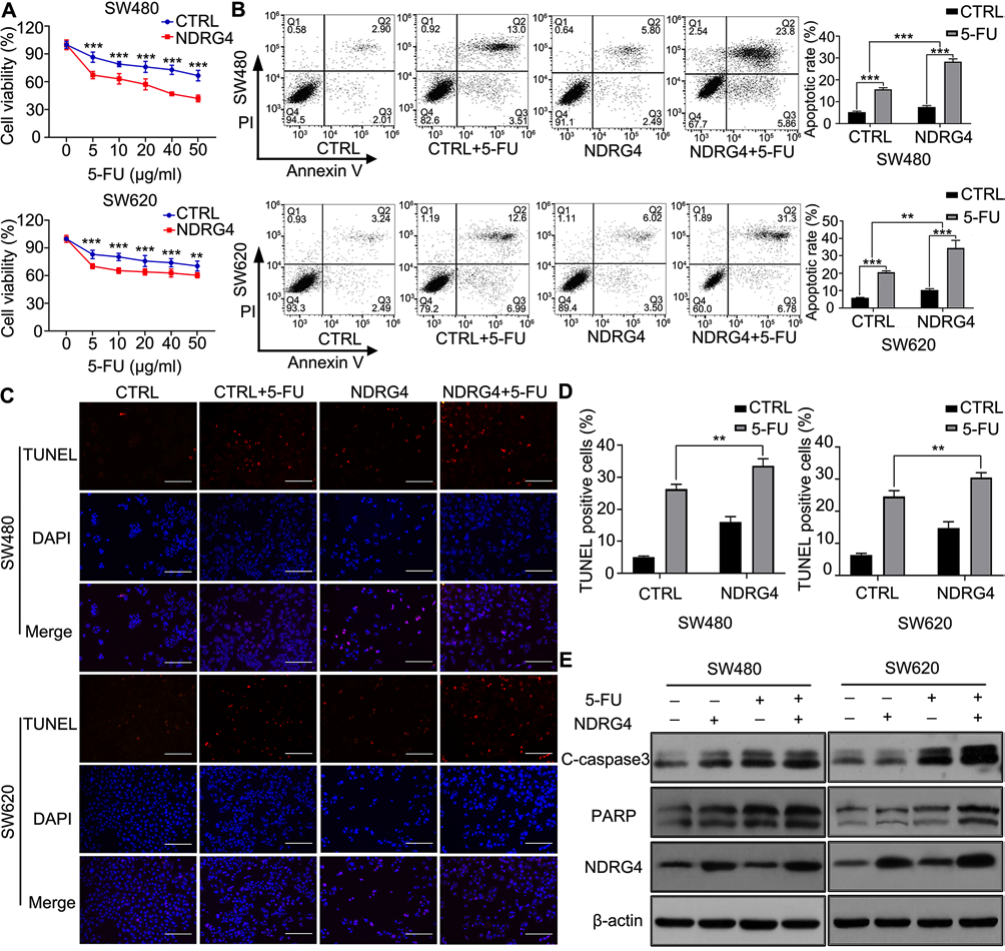
NDRG4 promotes 5-FU-induced colorectal cancer cell apoptosis. (A) Viability of SW480 and SW620 cells after 48 h of culture with different concentrations of 5-FU, determined by MTT assay. Statistical analysis by two-way ANOVA (n=5). (B) Detection of apoptosis in SW480 and SW620 control and NDRG4-overexpressing cells with or without 5-FU by flow cytometry. Top: Representative flow cytometric images and statistical analysis of the apoptotic rate of each group of SW480 cells. Bottom: Representative flow cytometric images and statistical analysis of the apoptotic rate of each SW620 cell group. Statistical analysis by two-way ANOVA (n=3). (C) Detection of apoptosis in SW480 and SW620 control and NDRG4-overexpressing cells with or without 5-FU by TUNEL assay (n=3). Scale bar, 150 μm. (D) Statistical analyses of the TUNEL assay results by two-way ANOVA. (E) Apoptosis-associated protein expression in SW480 and SW620 control and NDRG4-overexpressing cells with or without 5-FU as determined by western blotting. Multiple bands of C-caspase3 represent the large fragment (17/19 kDa) of activated caspase-3 resulting from cleavage adjacent to Asp175. Upper band of PARP represents full-length PARP-1, and the lower band represents the large fragment produced by caspase cleavage at Asp214 (n=3). ***P<0.001 and **P<0.01 vs. CTRL. NDRG4, N-myc downstream-regulated gene 4; 5-FU, 5-fluorouracil; C-caspase, cleaved caspase; CTRL, control; PARP, poly-ADP-ribose polymerase; C-, cleaved.

